# ERO1L promotes NSCLC development by modulating cell cycle‐related molecules

**DOI:** 10.1002/cbin.11454

**Published:** 2020-09-15

**Authors:** Xiujuan Shi, Jiawen Wu, Yi Liu, Yuxiong Jiang, Changjiang Zhi, Jue Li

**Affiliations:** ^1^ Tongji University School of Medicine Shanghai China; ^2^ Institute of Clinical Epidemiology and Evidence‐Based Medicine Tongji University School of Medicine Shanghai China; ^3^ Key Laboratory of Arrhythmias of The Ministry of Education of China Tongji University School of Medicine Shanghai China; ^4^ Shanghai East Hospital Tongji University Shanghai China

**Keywords:** cell cycle, ERO1L, NSCLC, TCGA

## Abstract

Lung cancer is the leading cause of cancer‐related death worldwide. Previous studies revealed that endoplasmic reticulum oxidoreductase 1 alpha (ERO1L) played critical roles in the malignant behaviors of several cancer types, but its role in non‐small cell lung cancer (NSCLC) remained unclear. In this study, we identified 26 upregulated and 102 downregulated genes in NSCLC using bioinformatics analyses, and these genes were enriched in the biological processes of the cell cycle. ERO1L was remarkably upregulated in NSCLC and overexpression of ERO1L was associated with poor prognosis of NSCLC. ERO1L deficiency markedly suppressed NSCLC cell proliferation, colony formation, migration, and invasion. ERO1L depletion caused a dramatically decreased expression of cell cycle‐related factors in NSCLC cells. Collectively, our data validated that ERO1L could function as a tumor promoter in NSCLC, indicating the potential of targeting ERO1L for the treatment of NSCLC.

**Abbreviations**
ALK: anaplastic lymphoma kinase
EGFR: epidermal growth factor receptor
ERO1L: endoplasmic reticulum oxidoreductase 1 alpha
GEO: Gene Expression Omnibus
GO: Gene Ontology
KEGG: Kyoto Encyclopedia of Genes and Genomes
NSCLC: non‐small cell lung cancer
TCGA: The Cancer Genome Atlas

## 1 INTRODUCTION

Lung cancer is the leading cause of cancer death among men and the second leading cause of cancer death among women worldwide (Torre, Siegel, & Jemal, 2016). Approximately 80% of lung cancers are histopathologically classified as non‐small cell lung carcinomas (NSCLC; Mao, Yang, He, & Krasna, 2016). A plethora of genes have been verified to modulate NSCLC malignancies, such as EGFR, ALK, and BRAF (Dagogo‐Jack et al., 2019; Peters et al., 2017; Soria et al., 2018). Although the application of small molecule inhibitors and immunotherapy have contributed to unprecedented survival benefits in NSCLC patients, the treatment effects do not yet classify as prospective and the survival rates for NSCLC remain low (Hirsch et al., 2017). Hence, it is extremely necessary to identify novel biomarkers for better diagnosis and as more effective therapeutic targets to improve the outcomes in NSCLC.

Endoplasmic reticulum oxidoreductin‐1‐like protein (ERO1L; alias: ERO1A), located on chromosome 14, is a hypoxia‐inducible endoplasmic reticulum‐resident oxidase, and contributed to the introduction of disulfide bonds into the newly synthesized proteins (Cabibbo et al., 2000). Recently, growing studies have illustrated the important regulatory roles of ERO1L in several cancer types. For example, ERO1L was found to be upregulated in gastric cancer tissues and its high expression was associated with poor prognosis (Seol et al., 2016). In addition, ERO1L could also promote pancreatic cancer cell progression (Han, Xu, Zhao, Xiong, & Liu, 2018). Besides this, ERO1L is remarkably correlated with reduced survival and poor prognosis and promotes hepatocellular carcinoma metastasis and angiogenesis (Yang et al., 2018). Therefore, ERO1L might be involved in modulating cellular functional behaviors such as cellular growth, apoptosis, and metastasis. However, the clinical and functional relevance of ERO1L and its molecular mechanisms underlying NSCLC tumorigeneses remain largely unclear.

In the present study, the clinical significance and functional roles of ERO1L in NSCLC were investigated. We first applied a comprehensive bioinformatics analysis to determine the differentially expressed genes (DEGs) in NSCLC. Subsequently, the expression and clinical survival of ERO1L in NSCLC were also investigated by bioinformatics analysis. Moreover, we investigated the functional roles of ERO1L by evaluating the cellular growth and metastasis of NSCLC cells. Finally, the molecular mechanisms by which ERO1L exerted its critical regulatory roles in NSCLC cells were also explored. Collectively, our results provided a useful biomarker for NSCLC and new insights into ERO1L‐mediated NSCLC tumorigenesis.

## 2 MATERIALS AND METHODS

### 2.1 Cell culture

NSCLC cells (A549, H1299) and 293 T cells were obtained from Shanghai Cell Bank (Xuhui, Shanghai, China). A549 cells and H1299 cells were cultured using RPMI‐1640 media (Hyclone, Logan, UT, USA), and 293 T cells were maintained by DMEM‐F12 media (Hyclone). Fetal bovine serum (FBS; 10%; Hyclone) was essential to culture the cells. These cells were cultured in an incubator with 5% CO_2_ at 37°C.

### 2.2 Recombination lentivirus generation and infection

To knockdown ERO1L, short hairpin RNA or small hairpin RNA (shRNA) lentivirus vectors specific targeting ERO1L (shERO1L‐1 and shERO1L‐2) were constructed by Viraltherapy Technology Corporation (Wuhan, Hubei, China). The shRNA vectors were cotransfected with three package plasmids (pRRE, pREV, pVSVG) from a lentivirus package system (Invitrogen, Pudong, Shanghai, China) into 293 T cells (60% confluence) using Lipofectamine 3000 reagents (Invitrogen) in accordance with the manufacturer's protocols. The recombination lentivirus was collected 72 h post‐transfection. Subsequently, the lentivirus solution (10 μl) was respectively added into A549 cells or H1299 cells. After 24 h, the media were changed and the cells were utilized for other experiments.

### 2.3 Real‐time polymerase chain reaction

Trizol reagents (Shinegene, Songjiang, Shanghai, China) were employed for total RNA extraction. Afterward, the complementary DNA was reversely transcribed from 2 μg total RNAs by applying PrimeScript RT Reagent kits (Takara, Dalian, Liaoning, China). Subsequently, the relative expressing levels of ERO1L were determined by TransStart Green qPCR SuperMix kits (TransGen, Haidian, Beijing, China). The quantitative polymerase chain reaction (qPCR) conditions were as follows: pre‐denaturation at 94°C for 30 s, followed by 41 cycles of denaturation at 94°C for 5 s, annealing at 60°C for 30 s and with a final extension step at 72°C for 5 min. The primers were listed as follows: forward ERO1L (5ʹ‐GCCAGGTTAGTGGTTACTTGG‐3ʹ) and reverse ERO1L (5ʹ‐GGCCTCTTCAGGTTTACCTTGT‐3ʹ); forward glyceraldehyde‐3‐phosphate dehydrogenase (GAPDH; 5ʹ‐GGAGCGAGATCCCTCCAAAAT‐3ʹ) and reverse GAPDH (5ʹ‐GGCTGTTGTCATACTTCTCATGG‐3ʹ).

### 2.4 Western blot analysis

RIPA reagents (ABSin, Pudong, Shanghai, China) were applied for protein extraction after the cells were washed by PBS. After the protein concentrations were measured, the proteins (25 μg) were loaded on the sodium dodecyl sulfate‐polyacrylamide gel electrophoresis (SDS‐PAGE) and separated, followed by being transferred to polyvinylidene fluoride membranes. Then, the membranes were washed three times using Tris‐buffered saline with Tween 20 (TBST) buffer, followed by being probed using primary antibodies. The antibodies and the membranes were incubated at 4°C overnight. On the second day, TBST buffer was utilized for washing the membranes, followed by treating the membranes with the corresponding secondary antibodies. Lastly, the proteins were visualized by ECL kits (Beyotime, Haimen, Jiangsu, China). The primary antibody against ERO1L was purchased from Abcam Corporation (Pudong, Shanghai, China). The anti‐cyclin D1 and anti‐CDK6 antibodies were purchased from Proteintech Group Company (Wuhan, Hubei, China).

### 2.5 Cell proliferation detection

The cell proliferation was determined by Cell Counting Kit‐8 (CCK‐8) assays using CCK‐8 kits (Beyotime). In short, shRNA lentivirus‐infected NSCLC cells were digested and washed using PBS. Then, the cells were placed into 96‐well plates at a density of 2000 cells per well. Thereafter, CCK‐8 reagents (12 μl/well) were placed into the cells. After 3 h incubation at 37°C, the absorbance at OD 450 nm was determined using a microplate reader.

### 2.6 Wound‐healing assay

The lentivirus‐infected cells were placed into 12‐well plates and cultured until the cell confluence reached 100%. Then, the cell monolayers were scratched by 10 μl pipette tips to generate wounds. The wounds were washed using PBS buffer to remove the dead cells and cell debris. Then, media was added into the cells and the pictures of the wounds were taken at 0 h. After 24 h, the closures of the wounds were also photographed.

### 2.7 Transwell invasion assay

The lentivirus‐infected cells were collected and placed into the upsides of the transwell inserts at a density of 1.5 × 10^5^ cells/well. The transwell inserts were precoated using Matrigel and 250 μl serum‐free media were added into the upper chambers. Then, the lower chambers were added with 750 μl media containing 15% FBS. Forty‐eight hours later, the invaded cells were fixed using paraformaldehyde (4%) and treated by crystal violet solution (0.1%). After treatment for 15 min, the cells were washed using PBS and photographed using a Nikon microscope (TE2000; Nikon, Tokyo, Japan).

### 2.8 Microarray data and The Cancer Genome Atlas (TCGA) data

Microarray data of NSCLC tumor and normal tissues from four independent datasets (GSE18842, GSE19188, GSE30219, and GSE32863) were obtained from Gene Expression Omnibus (GEO, https://www.ncbi.nlm.nih.gov/geo/). RNA‐seq data of lung adenocarcinoma (LUAD) tumor specimens (502) and matched normal control samples (49) were downloaded from TCGA.

### 2.9 Identification of DEGs

The edgeR packages of Bioconductor analysis tools were used to identify the DEGs in NSCLC tumor samples and corresponding noncancerous tissues, using FDR < 0.05 and |log FC| ≥ 1 as cut‐off criteria. The common DEGs in the four datasets (GSE18842, GSE19188, GSE30219, and GSE32863) were screened out by Venny 2.1 (https://bioinfogp.cnb.csic.es/tools/venny/).

### 2.10 GO function and KEGG pathway enrichment analysis, and protein–protein interaction (PPI) network

Gene Ontology (GO) and Kyoto Encyclopedia of Genes and Genomes (KEGG) enrichment were analyzed by clusterProfiler R package (*p*‐adj < .01). PPI network of common DEGs was constructed by the latest STRING v10.5 database and visualized by Cytoscape v3.3.0 software.

### 2.11 Online database

Immunochemical staining analysis of ERO1L and CDH3 was conducted using the Human Protein Atlas (http://www.proteinatlas.org/). The expression of ERO1L in NSCLC patients' race, gender, age, smoking habits, and nodal metastasis; the expression of CDH3 and FAM83A; and the promoter methylation levels of these genes were analyzed using the UALCAN algorithm (http://ualcan.path.uab.edu/; Chandrashekar et al., 2017). Gene expression was also analyzed by the GEPIA (Gene Expression Profiling Interactive Analysis) algorithm (http://gepia.cancer-pku.cn/; Tang et al., 2017). Overall survivals were analyzed by the GEPIA algorithm (http://gepia.cancer-pku.cn/) or Kaplan–Meier plotter website (http://www.kmplot.com/; Nagy, Lanczky, Menyhart, & Gyorffy, 2018). The expression and overall survival of genes across diverse cancer types were also analyzed by the GSCALite algorithm (http://bioinfo.life.hust.edu.cn/web/GSCALite/; Liu et al., 2018).

### 2.12 Statistical analyses

The statistical analyses were performed with the software of SPSS (SPSS Inc., Chicago, IL, USA). All the data were expressed as mean ± *SEM*. The statistical significance was determined by analysis of variance (ANOVA) or two‐tailed *t test*, and the results were considered significant at a *p* < .05.

## 3 RESULTS

### 3.1 Bioinformatics analyses of DEGs in NSCLC

**Figure 1:**
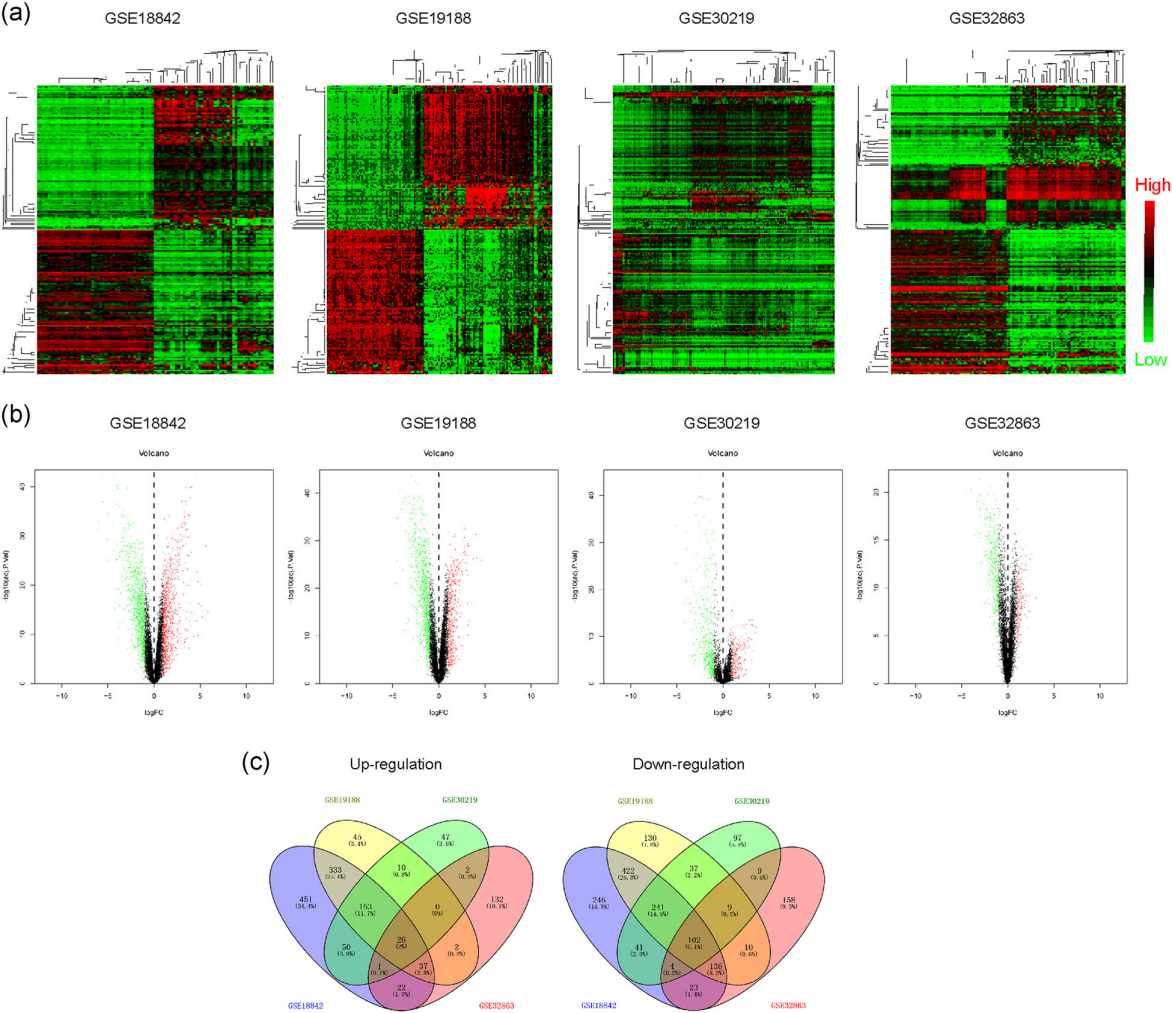
The DEGs in NSCLC were analyzed using GEO data. (a) Heatmaps of DEGs in GSE18842, GSE19188, GSE30219, and GSE32863. The red color represented the highly expressed genes in tumor tissues and the green color represented the low expression genes. (b) Volcano maps of DEGs in GSE18842, GSE19188, GSE30219, and GSE32863. (c) Venn diagrams of up‐ or downregulated genes in the above four microarray datasets. The Venn diagrams were generated by using Venny 2.1 database. DEGs, differentially expressed genes; GEO, Gene Expression Omnibus; NSCLC, non‐small cell lung cancer

To reveal the DEGs in NSCLC, we first conducted bioinformatics analyses using R packages. The microarray data (GSE18842, GSE19188, GSE30219, and GSE32863) were downloaded from GEO datasets and subsequently analyzed. The 200 most highly expressed and downregulated genes in each profile were subjected to draw heatmaps, and these heatmaps were shown in Figure 1a. The volcano maps of these microarray data are presented in Figure 1b. We then attempted to discover the commonly expressed genes across these microarrays. To achieve that, we intersected the analyzed results of these microarrays and performed Venn diagram analyses. The results figured out that there were 26 genes commonly upregulated and 102 genes commonly downregulated in these microarray data (Figure 1c and Table 1). Additionally, the expression of these 26 commonly upregulated genes across diverse cancer types was also analyzed using the GSCALite algorithm and we found that the majority of them were also highly expressed in various cancer types (Figure S1). Therefore, we obtained DEGs in NSCLC and these genes might play important roles in NSCLC development and progression.

**Table 1:** Differentially expressed genes

Upregulated genes	Downregulated genes
GPX2, CDC20, ASPM	ADH1B, AGER, CLIC5, FABP4, CPB2, FCN3, CYP4B1, FOSB, AGR3, FMO2, C7, C4BPA, CACNA2D2, FAM107A, EMCN, GLDN
CXCL14, DSP, CCNB2	FHL1, CFD, GPX3, CD52, CLIC3, CA4, CAV1, GIMAP8, CDO1, ANXA3, C1orf162, CAV2, FOXF1, ADRB2, FCN1, FGR, DPEP2, GADD45B, ABCA3, CYYR1, CDH5, GNG11, GDF10, AQP1, GIMAP7, FBLN5, CAMK2N1, AMICA1, CPA3, CCL2, ESAM, FOS
COL10A1, CTHRC1	CLDN5, CLEC14A, ALOX5AP, CD300LF, EGR1, C14orf132, COX7A1, GMFG, CA2, C5AR1, EPAS1, CD97, DUSP1, CYP27A1
CDCA7, CDH3, ECT2	DPYSL2, CALCRL, CLEC12A, EMP1, C1QB, EVI2B, ADAMTS1
CEACAM5, AURKA	CD37, C1QA, GIMAP4, GYPC, CLEC1A, CAT, AQP9, FCGR3B
AURKB, CDCA5, COL5A2	FCER1G, CBX7, CH25H, CYB5A, C1orf115, ALDH2, ARHGEF6
COL3A1, CRABP2, GPT2	BTK, C1orf21, FXYD6, ACVRL1, C20orf85, FZD4, FCGR1B, C9orf24, CCL23, CXCL2, EMP3, CTNNAL1, GPC3, EGR2, FMO3
CST1, F12, FAM83A	
COL17A1, ERO1L	C10orf54, FAM65A, AHNAK
COL1A1, CCNF	

### 3.2 Functional analysis of DEGs in NSCLC

**Figure 2:**
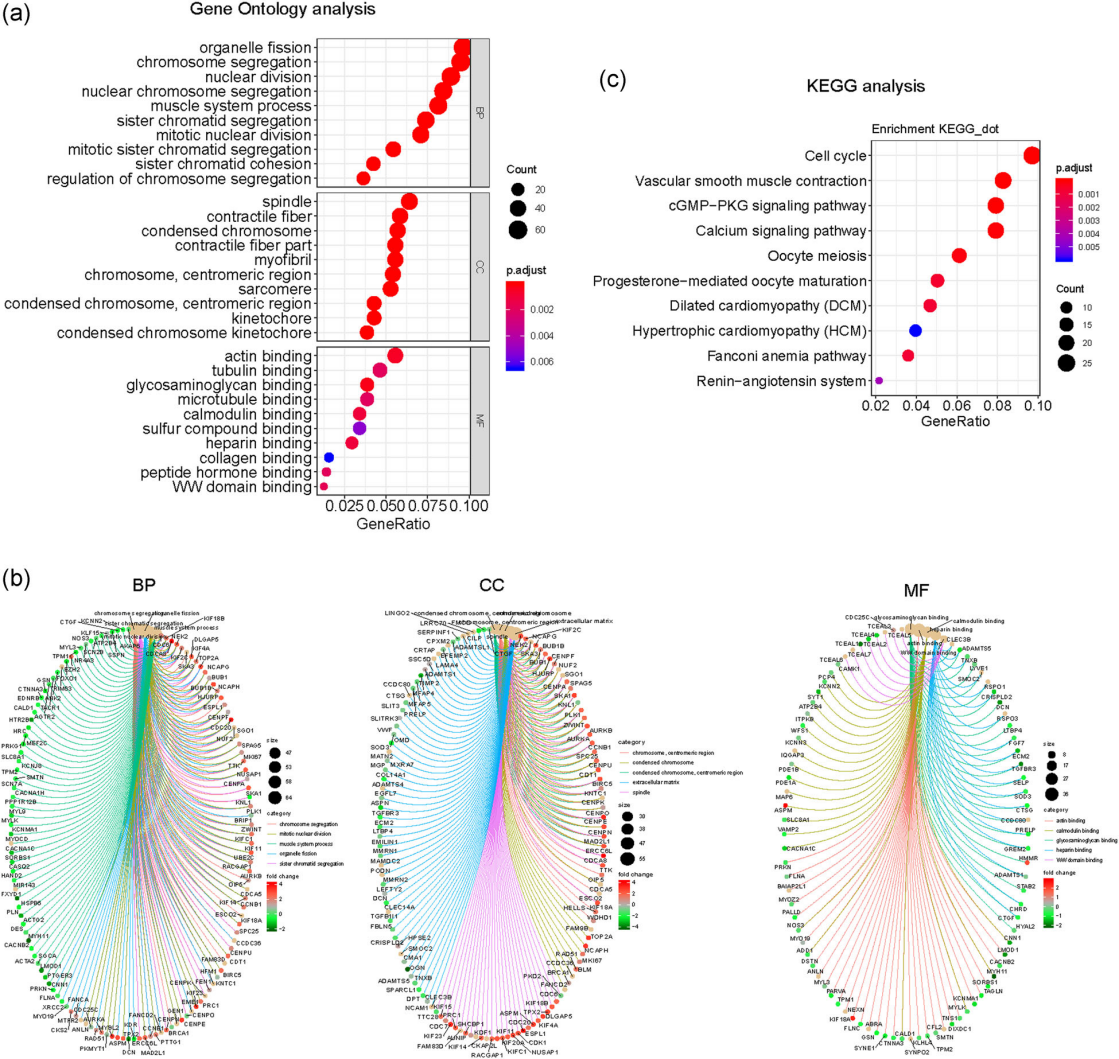
GO and KEGG analysis of the differentially expressed genes in NSCLC. (a) Bubble chart of GO analysis. (b) Circle diagram of the GO analysis. (c) KEGG analysis. BP, biological progress; CC: cell component; GO, Gene Ontology; KEGG, Kyoto Encyclopedia of Genes and Genomes; MF, molecular function; NSCLC, non‐small cell lung cancer

As we obtained DEGs in NSCLC, we next sought to investigate whether these DEGs played roles in functional regulation in NSCLC. Therefore, we conducted functional analysis of these DEGs using bioinformatics analyses. GO enrichment analyses of the DEGs were first carried out and the top 10 GO terms in three classical groups (BP, biological progress; CC, cell component; MF, molecular function) were presented using the bubble diagram analyses. The results elucidated that these commonly up‐ and downregulated genes were relevant with cell division‐related GO categories, such as “nuclear division,” “spindle,” and “microtube binding,” indicating that these genes possibly affected cell proliferation (Figure 2a). Moreover, circle diagram analyses were also conducted and further validated the results of the GO analyses (Figure 2b). In addition, KEGG pathway analyses demonstrated that these genes were remarkably related with the cell cycle (Figure 2c). Besides this, the interaction map of these 26 upregulated genes and pathways across diverse cancer types was also generated using the GSCALite algorithm (Figure S2). In addition, the interacting networks among these DEGs were also generated by STRING analyses (Figure S3). Taken together, the functional investigation indicated that these DEGs were possibly relevant to NSCLC malignancies.

### 3.3 Expressing validation by TCGA datasets

**Figure 3:**
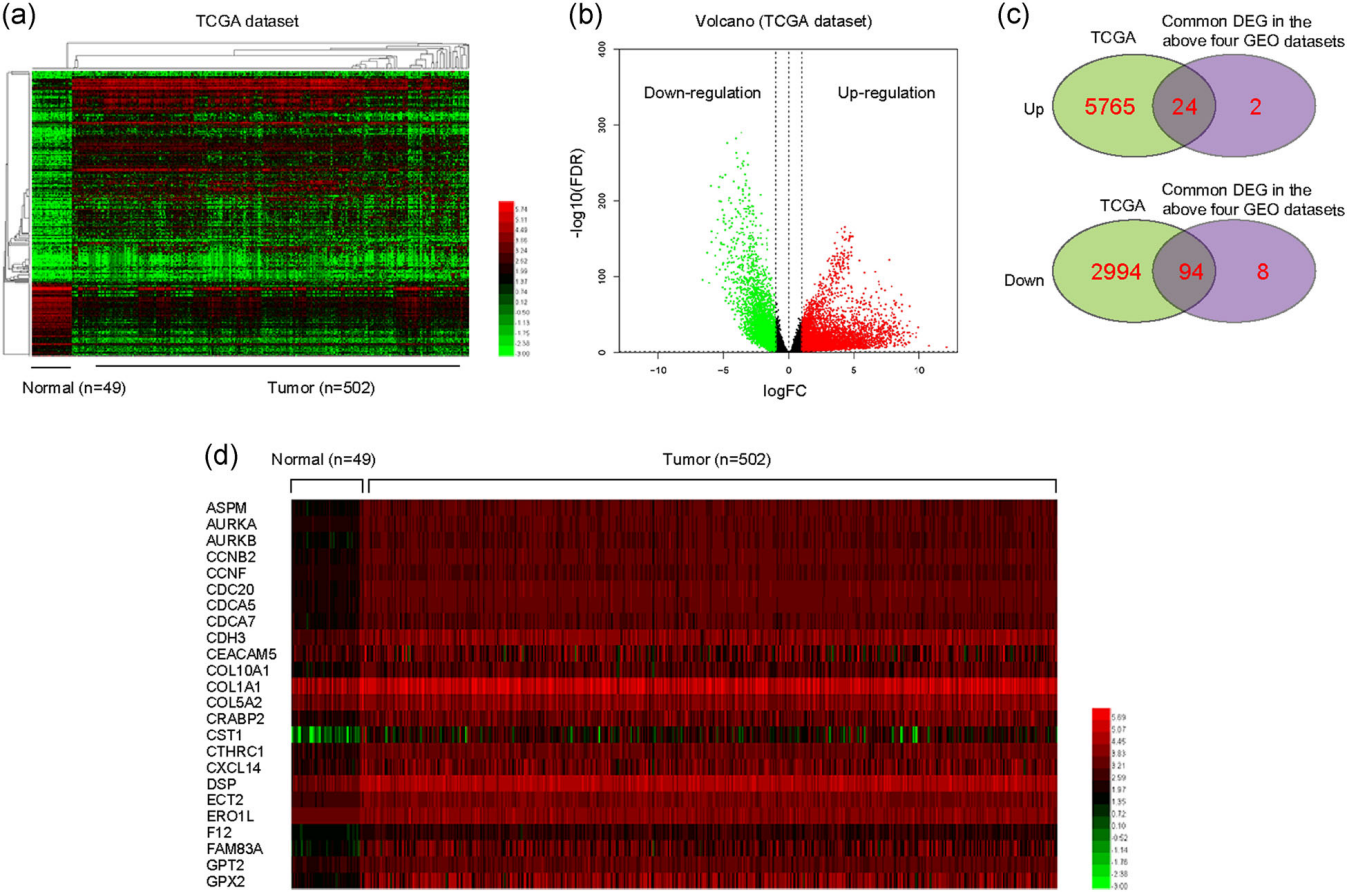
The DEGs in NSCLC were analyzed using TCGA data. (a) Heatmaps of DEGs in TCGA data. The red color represented the highly expressed genes in tumor tissues and the green color represented the low expression genes. (b) Volcano map of DEGs in TCGA data. (c) Venn diagrams of up‐ or downregulated genes in the above analysis. (d) The heatmap of these 24 genes expression in NSCLC (using TCGA data). DEGs, differentially expressed genes; GEO, Gene Expression Omnibus; NSCLC, non‐small cell lung cancer; TCGA, The Cancer Genome Atlas

We next aimed to investigate whether the above DEGs were also upregulated in NSCLC tumor samples using TCGA dataset analyses. Hence, we analyzed the NSCLC TCGA dataset and obtained thousands of DEGs. The heatmaps and volcano maps are presented in Figure 3a,b. We then intersected these DEGs in the TCGA dataset and the above four GEO datasets. The results suggested that there were 24 upregulated common genes and 94 downregulated common genes (Figure 3c). As mounting evidence had implied that highly expressed genes were closely relevant with tumor malignant behaviors, we next focused on these 24 upregulated genes. The heatmap of these 24 genes expression in NSCLC was presented in Figure 3d, and the heatmap clarified that these 24 genes were indeed upregulated in NSCLC tumor samples. Collectively, these analyses further confirmed that these 24 upregulated genes were indeed highly expressed in NSCLC tumor specimens.

### 3.4 Clinical significance analyses of the upregulated genes

**Figure 4:**
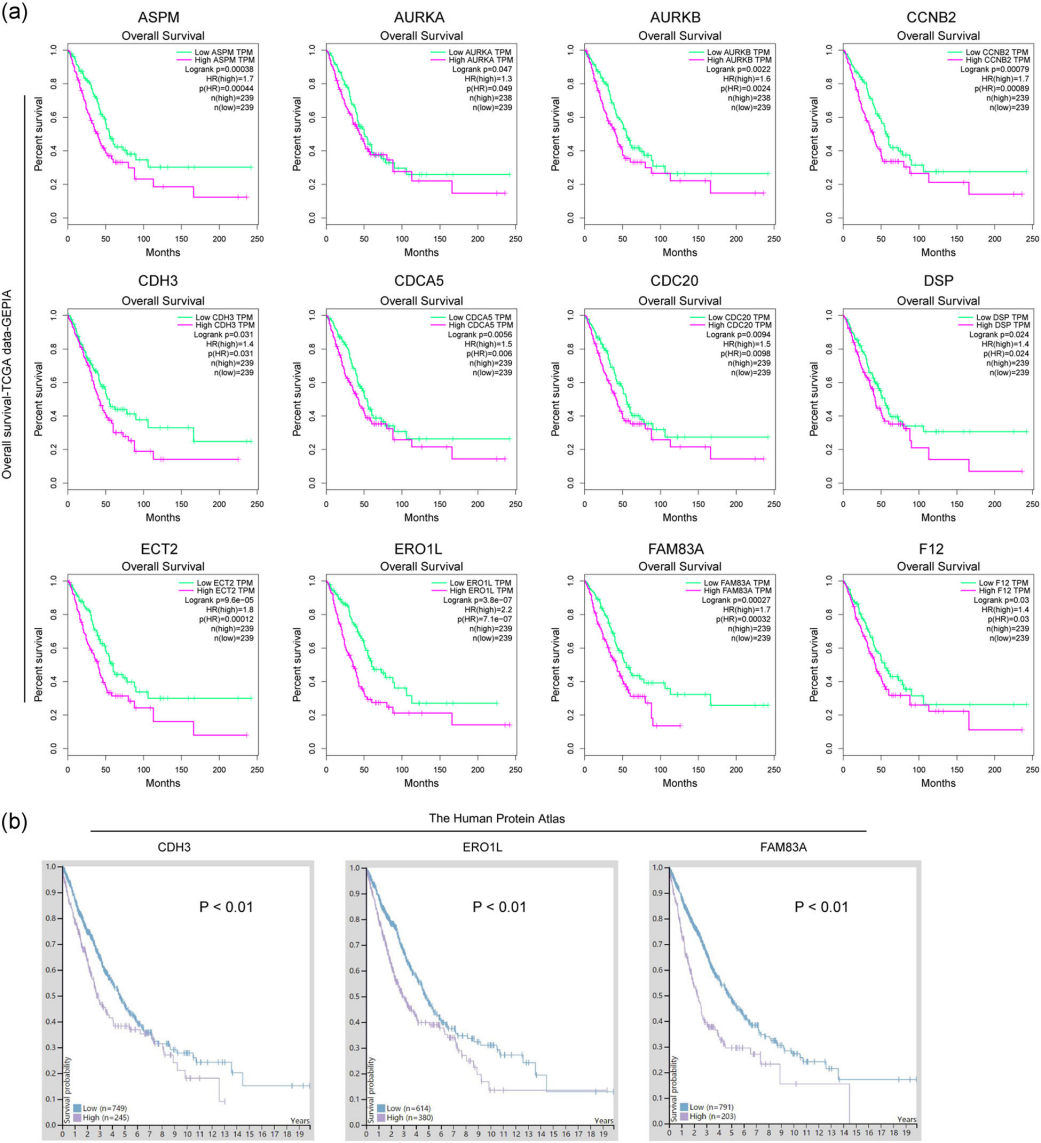
Overall survival analysis. (a) Overall survival analysis using GEPIA algorithm. The data (mRNA expressing levels) were based on TCGA dataset. The picture only presented overall survival analysis of the genes which were significantly correlated with overall survival curves of NSCLC patients. The green line represented the low gene expression in tumor tissues and the purple line represented the high gene expression in cancer tissues. (b) Overall survival analysis of CDH3, ERO1L, and FAM83A by using the protein‐expressing data from Human Protein Atlas dataset. GEPIA, Gene Expression Profiling Interactive Analysis; mRNA, messenger RNA; NSCLC, non‐small cell lung cancer; TCGA, The Cancer Genome Atlas

We next sought to explore whether these 24 commonly upregulated genes were correlated with significant overall survival of NSCLC patients. Using the GEPIA algorithm, we found that only a half of these genes were significantly correlated with the overall survival curves of NSCLC patients (Figure 4a). Interestingly, survival curves analyses across multiple cancer types revealed that these genes were also associated with several other cancer types, especially kidney‐related cancer types (Figure S4). In addition, the overall survivals of these genes were also analyzed by applying the Human Protein Atlas datasets, and the results showed that only three genes (ERO1L, CDH3, and FAM83A) were significantly associated with NSCLC patients' overall survivals (Figure 4b). Therefore, we discovered that upregulation of ERO1L, CDH3, or FAM83A was significantly correlated with poor prognosis in NSCLC patients.

### 3.5 Highly expressed ERO1L was associated with poor prognosis of NSCLC patients

**Figure 5:**
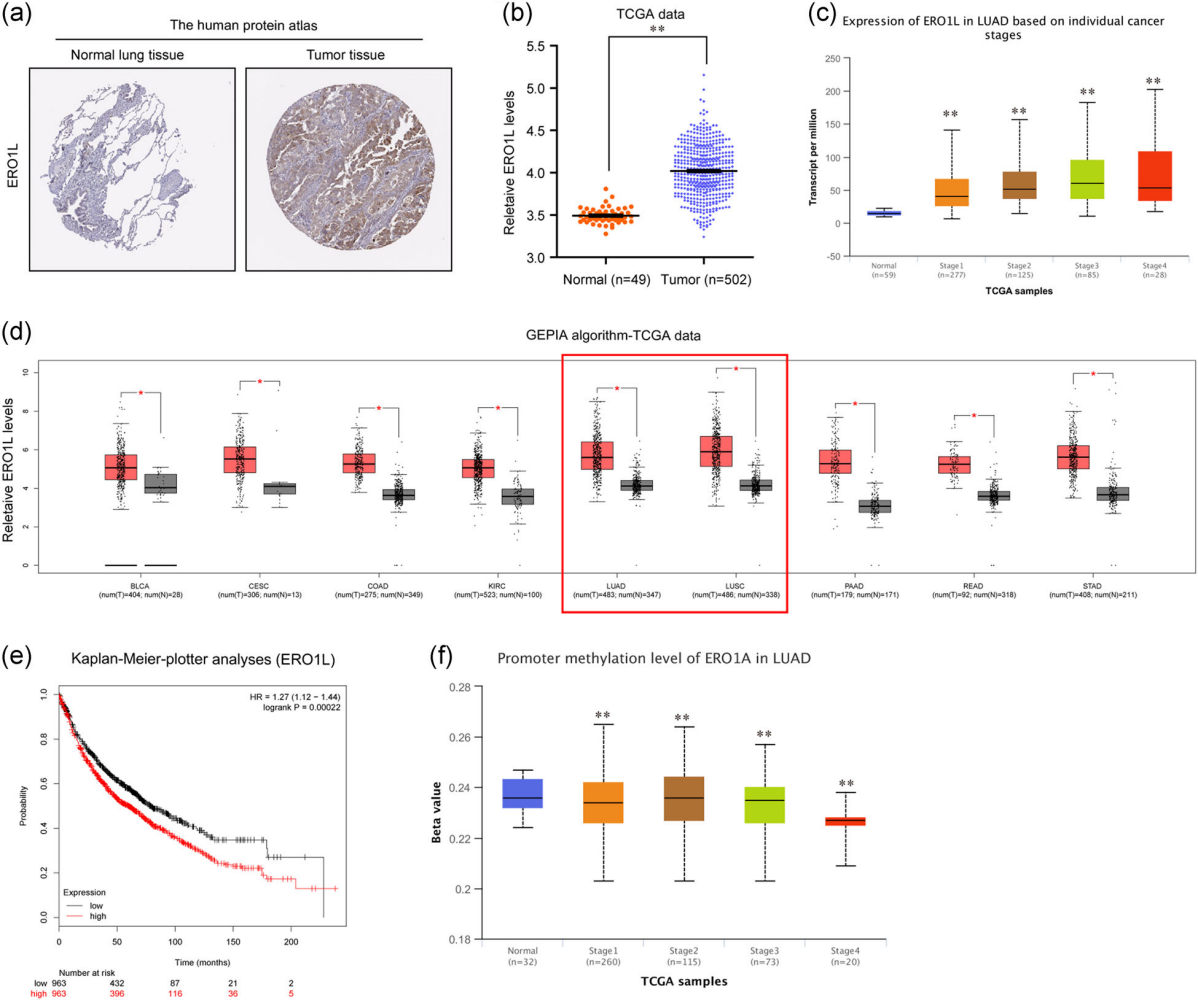
Overexpression of ERO1L was correlated with lower overall survival in NSCLC patients. (a) Immunochemical staining analysis of ERO1L by the Human Protein Atlas dataset. (b) The mRNA levels of ERO1L in NSCLC were analyzed by using TCGA data. (c) ERO1L levels across NSCLC stages I–IV were analyzed by the UALCAN algorithm. (d) ERO1L levels across diverse cancer types were analyzed by GEPIA algorithm. (e) Overall survival analysis by Kaplan–Meier Plotter website. (f) The promoter methylation of ERO1L in NSCLC samples (from stages I to IV) was analyzed by the UALCAN algorithm. *p < .05 and **p < .01. ERO1L, endoplasmic reticulum oxidoreductase 1 alpha; GEPIA, Gene Expression Profiling Interactive Analysis; LUAD, lung adenocarcinoma; mRNA, messenger RNA; NSCLC, non‐small cell lung cancer; TCGA, The Cancer Genome Atlas

Among the three genes (ERO1L, CDH3, or FAM83A), ERO1L attracted our attention. Hence, we next attempted to analyze the expression and clinical significance of ERO1L in detail. Immunochemical staining analysis by the Human Protein Atlas suggested that ERO1L was remarkably upregulated in NSCLC tumor specimens (Figure 5a). The mRNA levels of ERO1L in NSCLC TCGA tumor samples were also of high expression (Figure 5b). Furthermore, its upregulated expression was also confirmed by applying the UALCAN algorithm, and we found that ERO1L levels were elevated across NSCLC stages I–IV (Figure 5c). In addition, the expression of ERO1L in NSCLC patients' race, gender, age, smoking habits, and nodal metastasis was also analyzed using the UALCAN algorithm (Figure S5a‐e). GEPIA algorithm showed that ERO1L was upregulated in many malignant cancer types (Figure 5d). Kaplan–Meier plotter analyses also validated that the high expression of ERO1L was strongly associated with poor prognosis of NSCLC patients (Figure 5e). As mounting evidence had demonstrated that promoter methylation status was strongly relevant with gene expression, we next aimed to analyze the promoter methylation of ERO1L in NSCLC samples using the UALCAN algorithm, and the results certified that the promoter methylation was markedly decreased along with NSCLC progression from stages I–IV, which was consistent with the observation of that ERO1L levels that were elevated across NSCLC stages I–IV (Figure 5f). Besides this, the promoter methylation levels of ERO1L in NSCLC patients' race, gender, and age were also analyzed using the UALCAN algorithm (Figure S6a‐c). Hence, these data further demonstrated that ERO1L levels were upregulated in NSCLC tumor samples and might be a potential target of NSCLC therapy.

### 3.6 Repressing ERO1L expression abrogated malignancies of NSCLC in vitro

**Figure 6:**
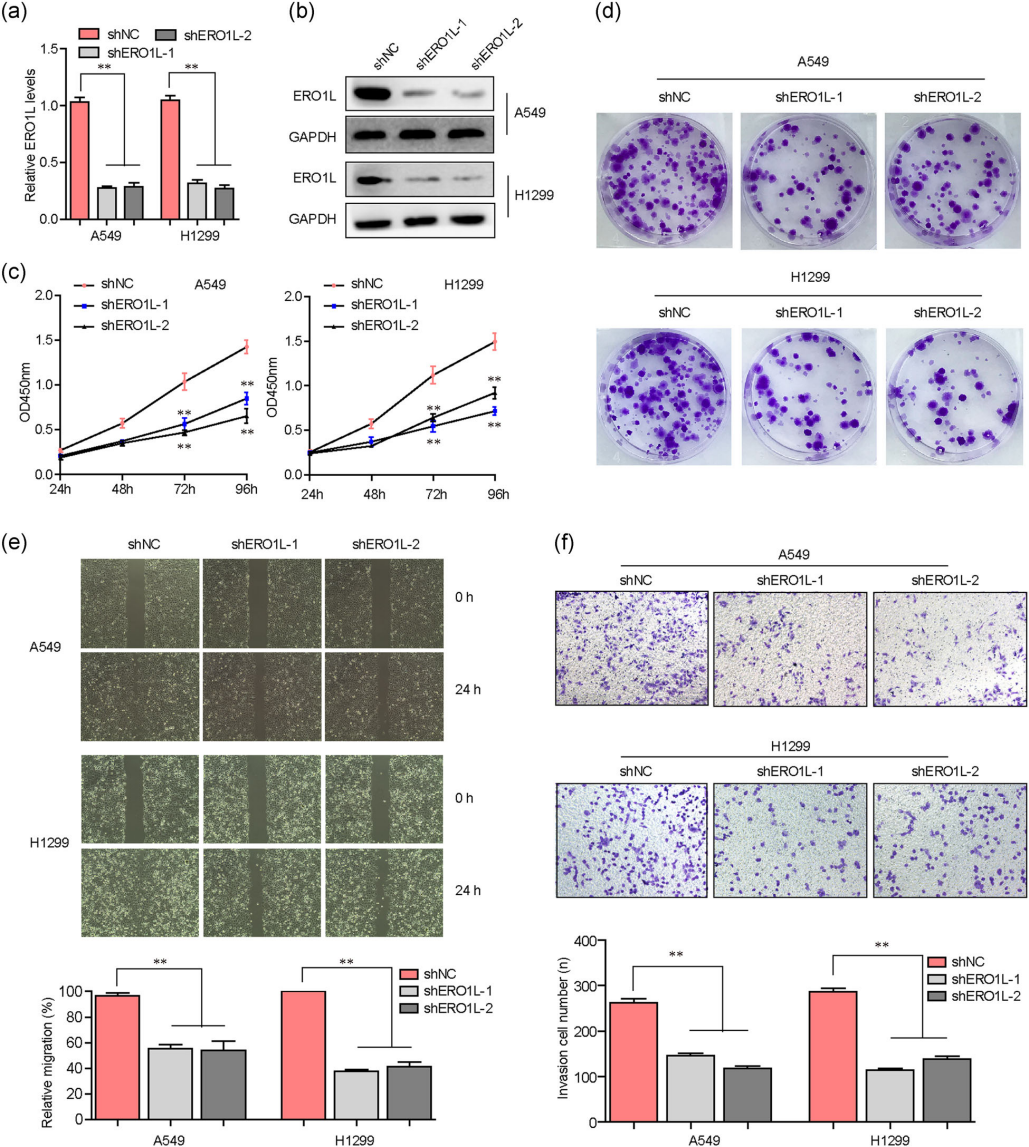
ERO1L regulated the malignant behaviors of NSCLC cells. (a) qPCR detected the mRNA levels of ERO1L in NSCLC cells after silencing ERO1L. (b) Western blot analysis detected ERO1L protein levels in A549 and H1299 cells. (c) CCK‐8 assays were performed to determine the cell proliferation of NSCLC cells after ERO1L depletion. (d) Colony formation assays evaluated the colony formation abilities of NSCLC cells. (e) Wound‐healing assays examined the migration of NSCLC cells. (f) Transwell assays determined the invasion of NSCLC cells. *p < .05 and **p < .01. CCK‐8, Cell Counting Kit‐8; ERO1L, endoplasmic reticulum oxidoreductase 1 alpha; GAPDH, glyceraldehyde‐3‐phosphate dehydrogenase; mRNA, messenger RNA; NSCLC, non‐small cell lung cancer; qPCR, quantitative polymerase chain reaction; TCGA, The Cancer Genome Atlas

Next, we carried out loss‐of‐function studies to investigate whether ERO1L was able to modulate the malignant behaviors of NSCLC cells. To achieve that, we first constructed shRNA vectors against ERO1L (shERO1L‐1 and shERO1L‐2). Then, the vectors were respectively packaged into lentivirus and the lentivirus was used for infecting NSCLC cells. Afterward, qPCR and western blot analysis were separately applied for detecting the knockdown efficiency in NSCLC cells (Figure 6a,b). Subsequently, we performed CCK‐8 assays and the results demonstrated that ERO1L deficiency resulted in notably decreased cell proliferative rates of NSCLC cells (Figure 6c). Similar results were also observed from colony formation assays that repressing ERO1L expression caused remarkably decreased colony formation capabilities of NSCLC cells (Figure 6d). In addition, the influences of ERO1L on NSCLC cell metastasis were also investigated. Data from the wound‐healing assays revealed that the migration of NSCLC cells was markedly suppressed by ERO1L depletion (Figure 6e). Moreover, transwell assays validated that knocking down ERO1L expression contributed to a significant reduction of NSCLC cellular invasive abilities (Figure 6f). Collectively, the above findings suggested that ERO1L played critical roles in regulating NSCLC malignancies.

### 3.7 ERO1L knockdown inhibited the expression of cell cycle‐related molecules

**Figure 7:**
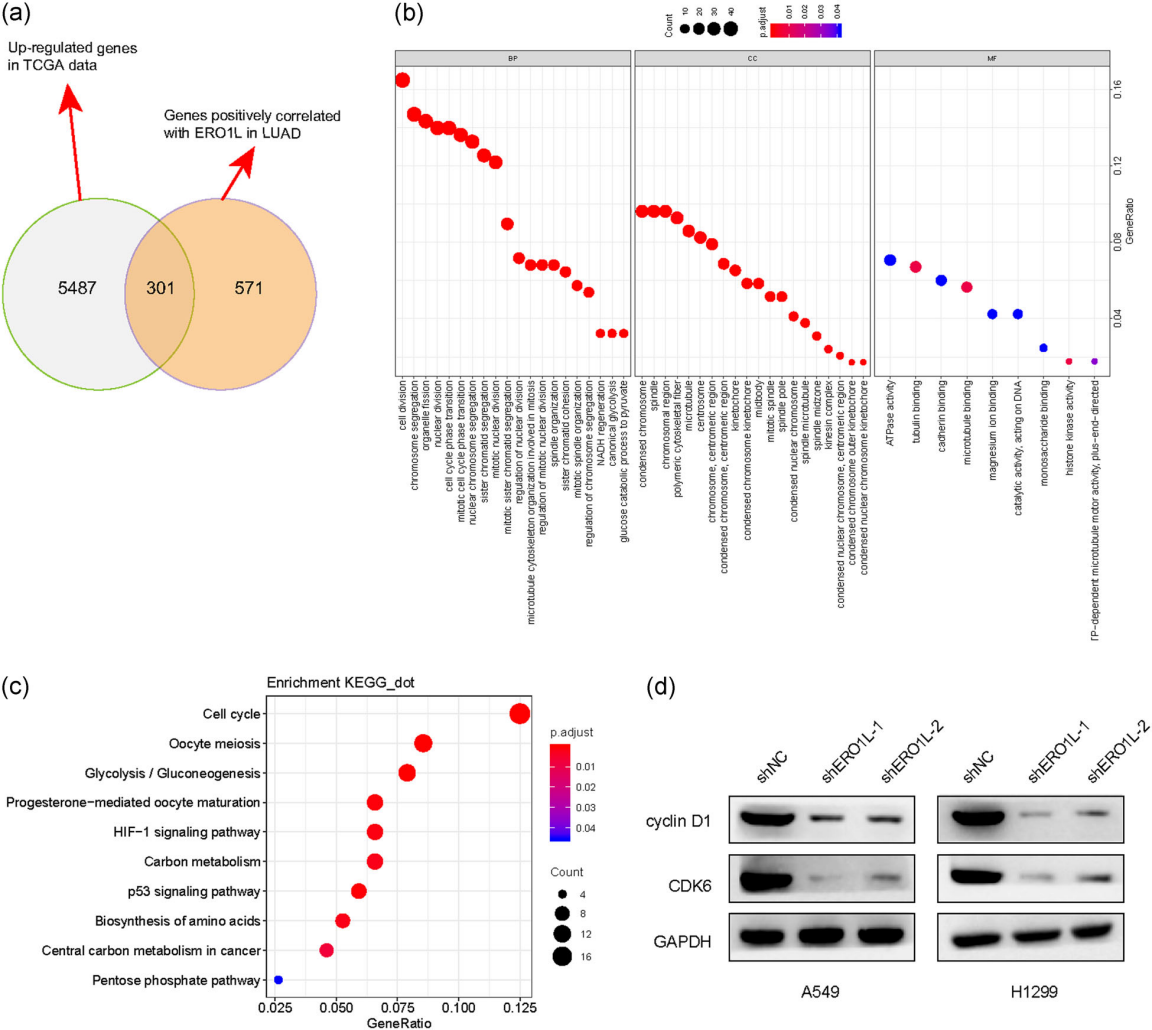
ERO1L modulated the levels of cell cycle‐related factors. (a) Venn diagram was utilized for drawing the intersection of upregulated genes in lung cancer‐TCGA data and genes positively correlated with ERO1L in lung cancer. The Venn diagrams were generated by using the Venny 2.1 database. (b) The 301 common genes obtained above were utilized for GO analysis. (c) The 301 common genes obtained above were utilized for KEGG analysis. (d) Western blot analysis measured the protein levels of cyclin D1 and CDK6 in NSCLC cells after ERO1L was depleted. (e) Flow cytometry was employed to measure the influence of ERO1L depression on NSCLC cell cycle. BP, biological progress; CC, cell component; ERO1L, endoplasmic reticulum oxidoreductase 1 alpha; GAPDH, glyceraldehyde‐3‐phosphate dehydrogenase; GO, Gene Ontology; KEGG, Kyoto Encyclopedia of Genes and Genomes; LUAD, lung adenocarcinoma; MF, molecular function; NSCLC, non‐small cell lung cancer; TCGA, The Cancer Genome Atlas

We next attempted to elucidate the mechanisms by which ERO1L modulates the malignant behaviors of NSCLC in detail. First, the genes positively correlated with ERO1L in NSCLC were obtained from the UALCAN algorithm. Then, these genes were intersected with upregulated genes in NSCLC TCGA data, and we found there were 301 common genes (Figure 7a). Subsequently, GO enrichment and KEGG analyses were conducted using these genes, and we found that they were closely relevant with the cell cycle (Figure 7b,c). Hence, these results indicated that ERO1L was associated with the cell cycle. We thereby employed western blot analysis to evaluate the levels of the key factors involved in cell cycle regulation in NSCLC cells after their ERO1L was depleted. The results validated that repressing ERO1L expression contributed to markedly decreased protein levels of cyclin D1 and CDK6 in NSCLC cells (Figure 7d). Therefore, ERO1L promoted NSCLC development through modulating cell cycle‐related molecules.

## 4 DISCUSSION

Until date, lung cancer remains the most frequent cause of cancer‐related deaths worldwide, and the 5‐year survival rates are still low (Hirsch et al., 2017). To improve the treatment of NSCLC, the identification of novel biomarkers and key molecules related to NSCLC metastasis are critical for NSCLC prognosis therapy (Duruisseaux & Esteller, 2018). In the past few decades, novel molecular targets were continuously discovered, prompting the development of new approaches for NSCLC (Camidge, Doebele, & Kerr, 2019). Particularly, the use of small‐molecule inhibitors such as tyrosine kinase inhibitors and immunotherapy had contributed to unprecedented survival benefits in NSCLC patients (Giroux Leprieur et al., 2017; Herbst, Morgensztern, & Boshoff, 2018). However, the overall survival rates for NSCLC remain low and the use of novel markers for diagnostic, or therapeutic approaches is still limited. Hence, the identification of novel dysregulated genes and continued research into new drugs would expand the clinical benefits to a broader patient population and improve outcomes in NSCLC. In this present study, we discovered the DEGs in NSCLC by analyzing both GEO data (GSE18842, GSE19188, GSE30219, and GSE32863) and TCGA data, and ERO1L was found to be one of the most highly expressed genes in NSCLC. In addition, we also found that ERO1L was upregulated in diverse cancer types using bioinformatics analysis. These findings were consistent with several previous studies that overexpression of ERO1L was also observed in gastric cancer (Zhou et al., 2017), oral squamous cell carcinoma (Li, Li, Jiang, Chen, & Gan, 2020) and lung adenocarcinoma (Hsu et al., 2016). Besides this, by applying bioinformatics analysis, we also found that high ERO1L expression was associated with lower overall survival in NSCLC patients. Therefore, these findings indicated that ERO1L overexpression might contribute to NSCLC progression.

ERO1L, playing critical roles in protein synthesis, had previously been found to serve as an oncogenic promoter in tumorigenesis of several cancer types. For example, overexpression of ERO1L was found to be relevant to poor prognosis of gastric cancer, and further functional studies confirmed that ERO1L depletion suppressed cell proliferation, migration, invasion, and chemoresistance (Seol et al., 2016). Moreover, after treatment with an inhibitor of ERO1L, EN‐460, the cellular growth of multiple myeloma was impaired and the cell apoptosis was promoted (Hayes et al., 2019). Additionally, ERO1L was also observed upregulated in breast cancer, and knockdown of ERO1L abrogated in vivo tumor growth and reduced metastasis (Kutomi et al., 2013; Tanaka et al., 2015). Therefore, these previous reports revealed that ERO1L played essential roles in the regulation of cancer progression. However, whether ERO1L also functioned as tumor promoter in NSCLC was unknown previously. Hence, in this study, we conducted functional studies to investigate this. Our data suggested that ERO1L deficiency markedly suppressed cell proliferation, colony formation abilities, migration and invasion of NSCLC cells, which was consistent with the results of the previous studies in other cancer types. Therefore, our findings not only enriched the knowledge of ERO1L in modulating cancer malignancies but also revealed that ERO1L might be a treatment target for NSCLC.

Although there were several expressing or functional studies of ERO1L, the molecular mechanism by which ERO1L exerted its regulatory functions in malignant behaviors was limited. Han et al. (2018) previously reported that ERO1L played critical roles in the progression of pancreatic cancer, and ERO1L was able to activate the Wnt/catenin pathway and upregulate the targets of this signaling. However, the molecular mechanisms by which ERO1L modulated the malignancies of NSCLC  were not investigated previously. Therefore, we first applied bioinformatics analysis to evaluate the potential molecular mechanisms of ERO1L in regulation of NSCLC progression, and we found that ERO1L was closely relevant to the cell cycle through KEGG pathway analysis. Afterward, the key factors involved in the regulation of the cell cycle were evaluated in NSCLC cells after their ERO1L was depleted. Many other reports had confirmed that cyclin D1 and CDK6 were critical regulators in the cell cycle, and the reduction of cyclin D1 and CDK6 led to a significant arrest at the G1‐phase (Sun et al., 2008; Tadesse, Yu, Kumarasiri, Le, & Wang, 2015). In the present study, our results suggested that ERO1L could modulate the expression of cell cycle‐related molecules: cyclin D1 and CDK6. Hence, we discovered another possible molecular mechanism of ERO1L regulating tumor development. Overall, ERO1L was an oncogenic promoter for NSCLC and it could promote NSCLC development through modulating cell cycle‐related molecules. ERO1L might be a novel potential target for NSCLC prognosis and therapy.

## CONFLICT OF INTERESTS

The authors declare that there are no conflict of interests.

## Supporting information

Supporting information.
