# Supplementary material for: ERO1L promotes NSCLC development by modulating cell cycle‐related molecules
Source: Cell Biol Int. 2020 Sep 15;44(12):2473–84. doi: 10.1002/cbin.11454 (PMC7692932; doi:10.1002/cbin.11454)
Supplement: Supplementary file 1 — Supporting information. [file CBIN-44-2473-s001.docx]

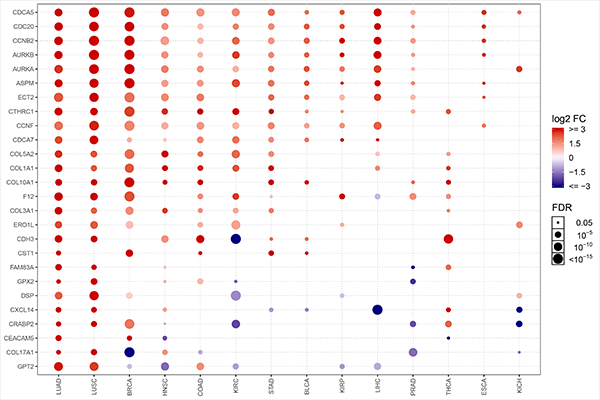


**Supplementary Figure 1.** The expression of 26 commonly up-regulated genes across diverse cancer types was analyzed using GSCALite algorithm


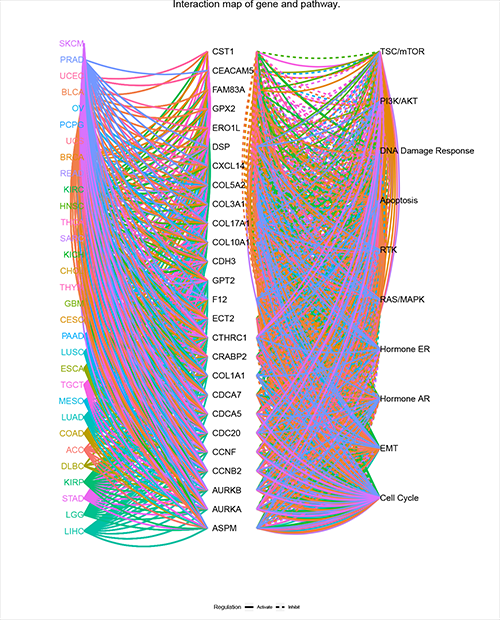


**Supplementary Figure 2.** The interaction map of these 26 commonly up-regulated genes and pathways across diverse cancer types was generated using GSCALite algorithm.


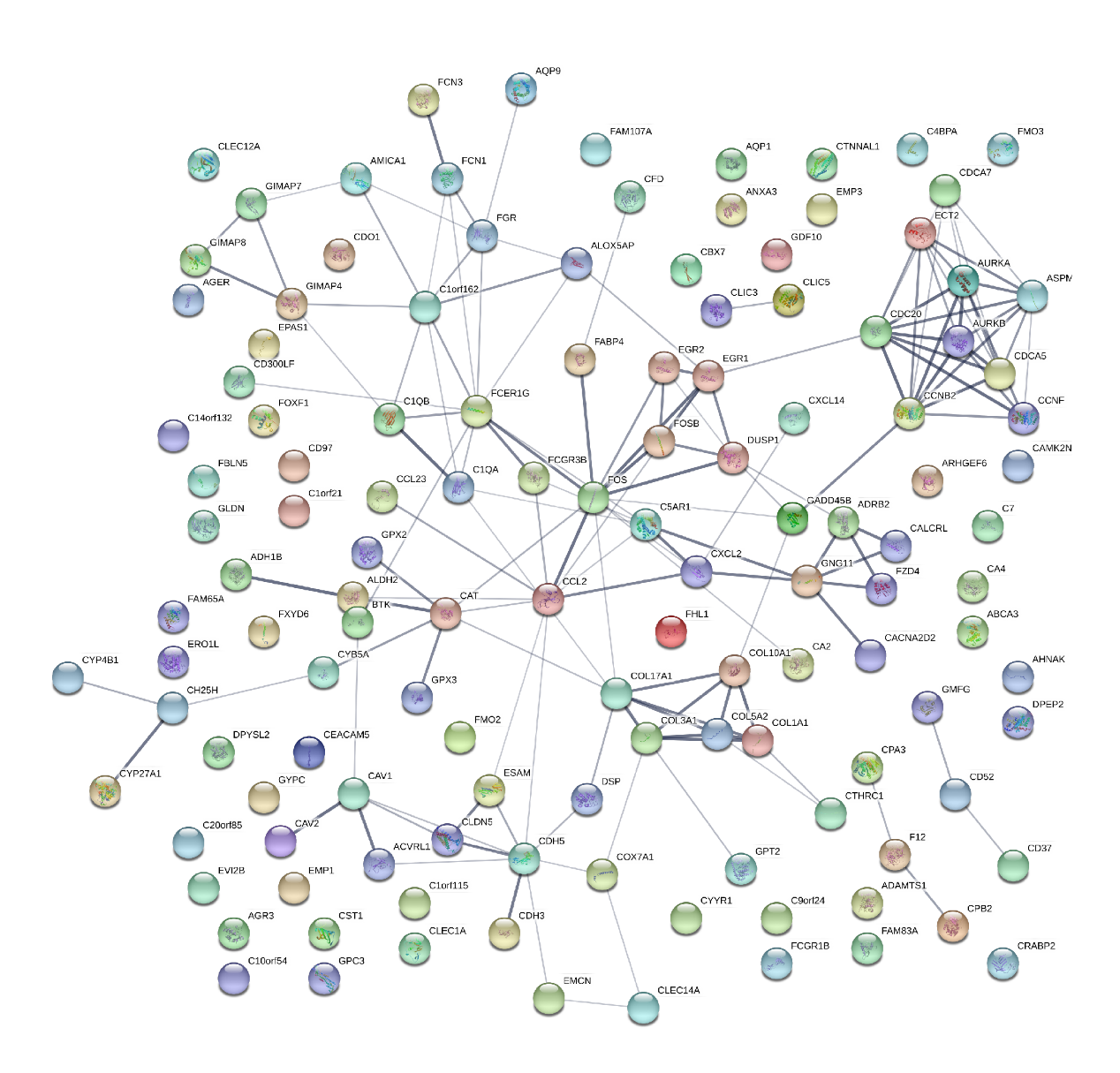


**Supplementary Figure 3.** The STRING program was applied for analyzing the protein-protein interaction (PPI) network.


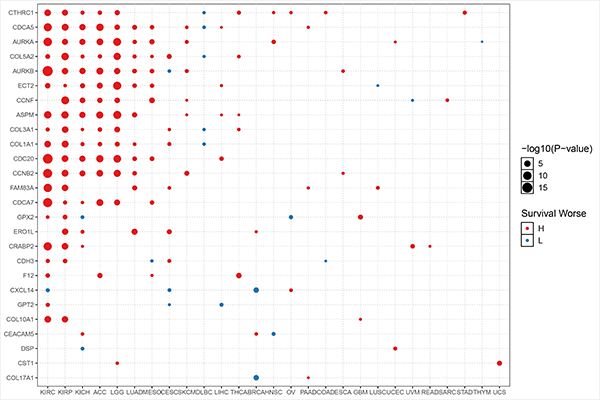


**Supplementary Figure 4.** Overall survivals of the corresponding gene set across multiple cancer types were analyzed by GSCALite algorithm.


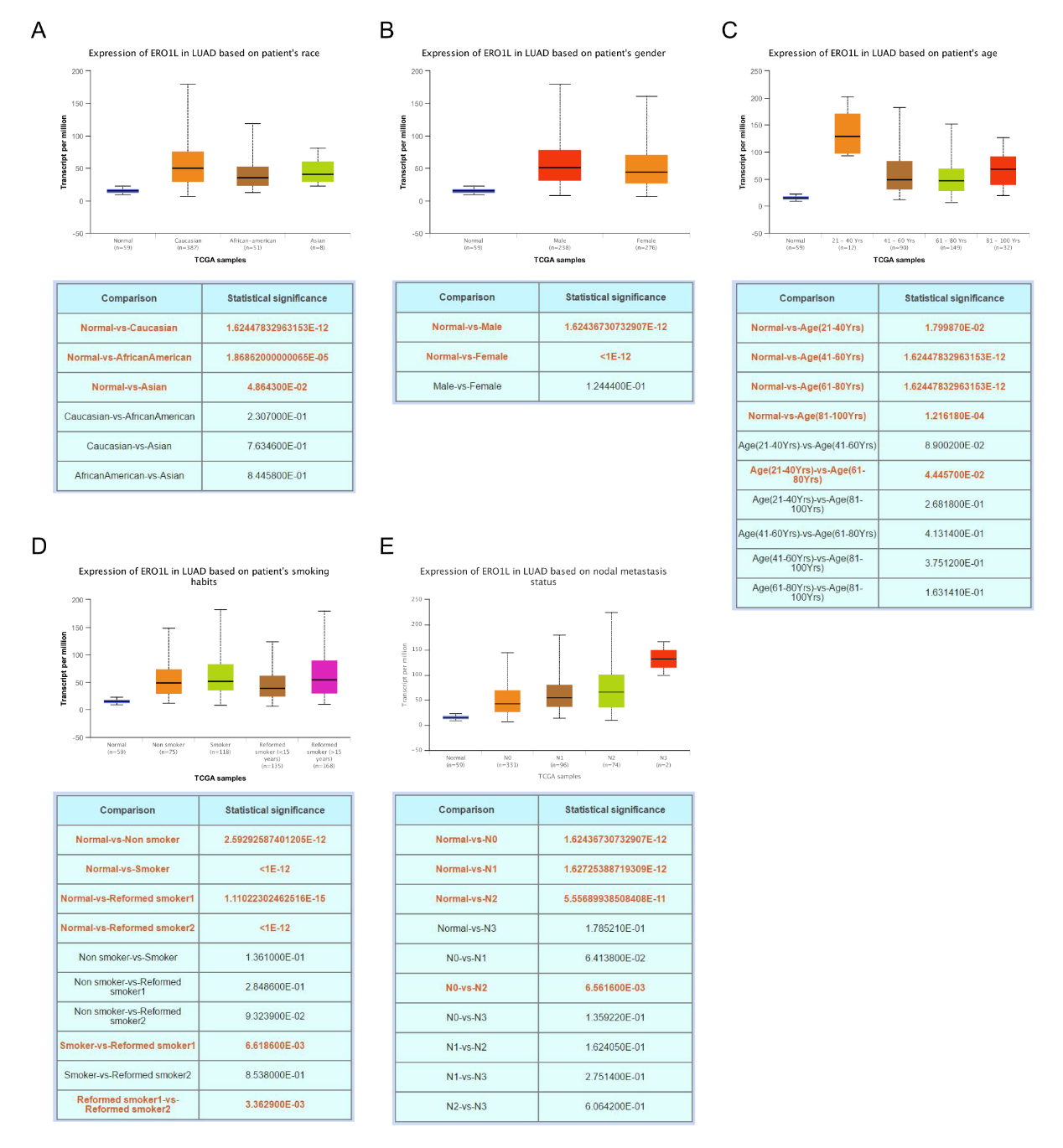


**Supplementary Figure 5.** The expression of ERO1L in NSCLC patients’ race (A), gender (B), age (C), smoking habits (D) and nodal metastasis (E) was also analyzed using UALCAN algorithm.


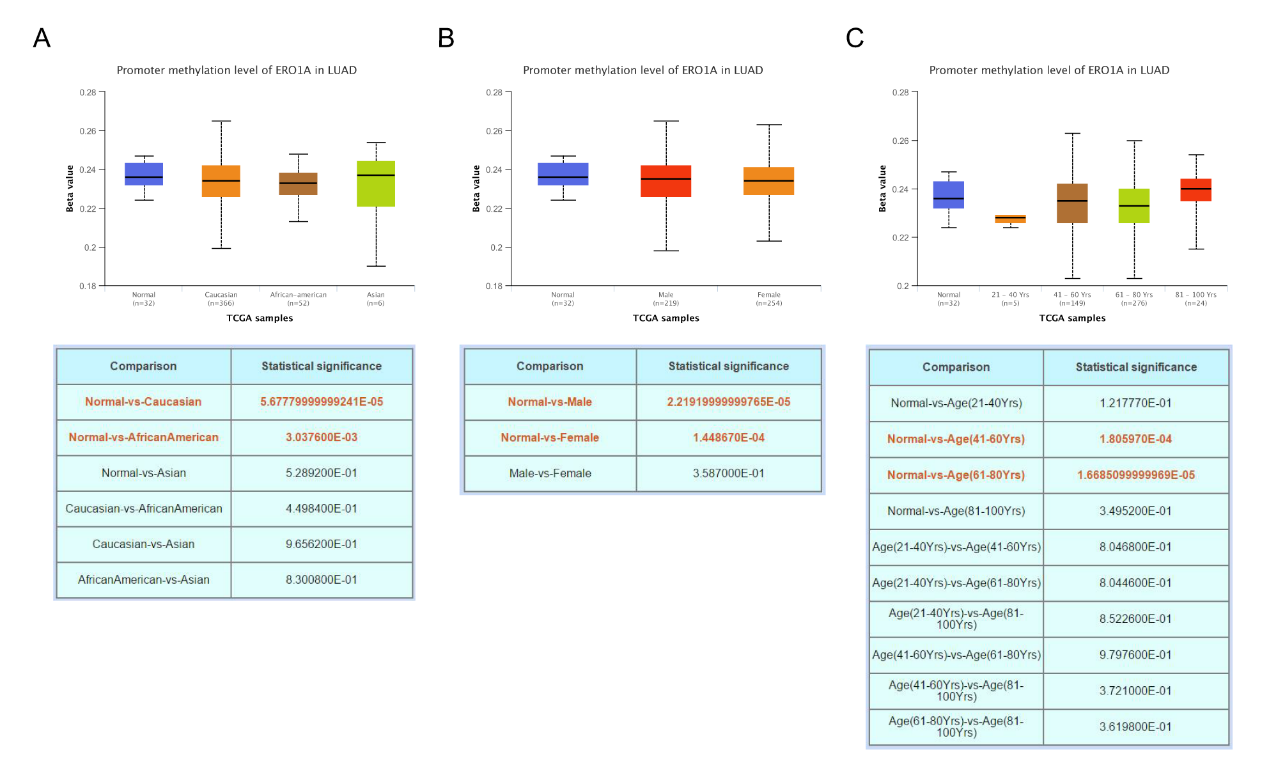


**Supplementary Figure 6.** The promoter methylation levels of ERO1L in NSCLC patients’ race (A), gender (B) and age (C) were also analyzed using UALCAN algorithm.
